# Popliteal venous thrombosis in juvenile arthritis with Baker cysts: report of 3 cases

**DOI:** 10.1186/1546-0096-6-12

**Published:** 2008-07-24

**Authors:** Frank Dressler, Cornelia Wermes, Eckart Schirg, Angelika Thon

**Affiliations:** 1 Department of Pediatrics, Medizinische Hochschule Hannover, Hannover, Germany; 2 Department of Radiology, Medizinische Hochschule Hannover, Hannover, Germany

## Abstract

Three pediatric patients with different illnesses leading to knee arthritis and large Baker cysts and additional calf swelling are reported. Calf swelling was due to true popliteal venous thrombosis and not to the much more common cause of pseudothrombophlebitis. Careful ultrasound examination can differentiate these two causes of calf swelling. Even though all our patients had risk factors for thrombophilia, we do not recommend routine thrombophilia work-up for all arthritis patients in the absence of thrombosis.

## 

Large Baker cysts-particularly following rupture-may lead to calf swelling and pseudothrombophlebitis that has been described in many adult and juvenile patients [1,2]. Pseudothrombophlebitis implies that symptoms of a venous thrombosis such as calf swelling are present, but not caused by thrombophlebitis. True popliteal venous thrombosis has only rarely been reported [3]. We recently saw 3 patients with different forms of juvenile arthritis and Baker cysts without a prior personal or family history of thrombotic events who developed popliteal venous thrombosis and upon work-up following the thrombosis had risk factors for thrombophilia.

Patient 1 was a 13-year old boy with a 9-year history of systemic juvenile idiopathic arthritis who developed left knee arthritis after a period of 18 months of disease remission off any medication. Several days later he also complained of painful calf swelling. On ultrasound examination a Baker cyst (52 × 28 × 24 mm) and 2 smaller cysts were found as well as incomplete popliteal venous thrombosis. During treatment with enoxaparin the thrombus dissolved within 2 weeks, and enoxaparin was stopped after 5 months. The knee arthritis improved with naproxen and methotrexate and after intraarticular injection of triamcinolone hexacetonide. At last follow-up 18 months later, the patient felt entirely well without effusion but a very small cyst remained.

Patient 2 was a 9-year-old girl with juvenile idiopathic oligoarthritis and 5 months of right knee arthritis treated with naproxen who newly complained of painful calf swelling. Sonography showed a 74 × 34 × 13 mm large Baker cyst as well as an incomplete thrombosis of the popliteal vein. With enoxaparin normal venous flow was reestablished within 3 months and enoxaparin was then stopped. The arthritis improved rapidly following intraarticular injection of triamcinolone hexacetonide and continued naproxen. Nine months later the girl had no complaints, and sonography found a smaller Baker cyst and a very small effusion.

Patient 3 was a 14-year old boy initially seen at another hospital with right knee arthritis. He was HLA-B27-positive and had highly positive IgG antibody titers against *Borrelia burgdorferi*. During 2 weeks of intravenous ceftriaxone his arthritis disappeared, but a few days after the end of antibiotic therapy he developed contralateral knee arthritis and calf swelling. Sonography found a large Baker cyst (86 × 12 mm) and complete popliteal venous thrombosis. He was treated with continuous intravenous heparin and switched to phenprocoumone within a few days, and his thrombosis disappeared within 6 weeks. He also received naproxen. When we first saw the patient 6 weeks after onset of the thrombosis, his arthritis had markedly improved, and the cyst could no longer be seen on ultrasound. Phenprocoumone and naproxen were both stopped 3 months after the onset of thrombosis, and another 13 months later the patient was asymptomatic and without evidence of joint effusion, cyst or thrombosis.

A broad investigation for risk factors for thrombophilia was performed in all 3 patients and risk factors found in our patients are shown in Table 1. Each patient had several risk factors. In particular, patient 2 had 2 known risk factors for venous thrombosis with heterozygous mutations in the prothrombin and the factor V genes. Other risk factors such as the PAI-1 (plasminogen activator inhibitor) and fibrinogen polymorphisms have been shown to increase the risks of stroke or myocardial infarction, but may have contributed to the thrombotic events in our patients. More common risk factors such as factor V Leiden, deficiencies in proteins C or S or antiphospholipid-antibodies were not present in our patients. The thrombophilia risk factors in our patients have not led us to restrict their physical activitites.

**Table 1:** Thrombophilia risk factors present (+) or absent (-) in our patients. Only those risk factors present in at least one of our patients are shown.

	Patient 1	Patient 2	Patient 3
Prothrombin G20210A mutation	-	+	-
Factor V A4070G mutation*	-	+	-
Lipoprotein (a) increase	+	+	-
Methylenetetrahydrofolatereductase (MTHFR) mutation**	+	+	+
PAI-1 4G/4G mutation	+	-	+
β fibrinogen G455A mutation	+	+	+
Lupus antibody^2^	+	-	-
Factor VIII increase^3^	-	+	+

All mutations were heterozygous except for the homozygous MTHFR mutation A1298C in patient 1.

*This is not the factor V Leiden mutation.

**MTHFR mutations were homozygous A1298C in patient 1, heterozygous A1298C in patient 2 and C677T in patient 3.

PAI = Plasminogen Activator Inhibitor.

^2^Lupus antibody was an IgM against beta-2-glycoprotein.

^3^Factor VIII increases were around 200% of the expected and present at the time of thrombosis, but may also be explained as acute phase reaction.

In conclusion, large Baker cysts can be mechanical factors contributing to popliteal vein thrombosis, at least in patients with risk factors for thrombophilia. Patients with large Baker cysts and calf swelling should receive careful ultrasound evaluation for the possibility of true thrombosis that could have been mistaken as pseudothrombophlebitis. Since thrombosis remains rare in patients with Baker cysts, we do not recommend routine thrombophilia work-up in the absence of thrombosis.

## Competing interests

The authors declare that they have no competing interests.

## Authors' contributions

FD and AT were the clinicians in charge of pediatric rheumatology patient care, CW performed the thrombophilia work-ups, ES did the ultrasound studies. All authors contributed to writing the manuscript with FD as the principal author.

## Consent

Written informed consent was obtained from the patient or where applicable the patient's parents, for publication of this Case report. A copy of the written consent is available for review by the Editor-in-Chief of this journal.
